# Fungal diseases as neglected pathogens: A wake-up call to public health officials

**DOI:** 10.1371/journal.pntd.0007964

**Published:** 2020-02-20

**Authors:** Marcio L. Rodrigues, Joshua D. Nosanchuk

**Affiliations:** 1 Instituto Carlos Chagas, Fundação Oswaldo Cruz (Fiocruz), Curitiba, Brazil; 2 Instituto de Microbiologia Paulo de Góes (IMPG), Universidade Federal do Rio de Janeiro, Rio de Janeiro, Brazil; 3 Department of Medicine (Division of Infectious Diseases) and Department of Microbiology and Immunology, Albert Einstein College of Medicine, New York, New York, United States of America; University of Tennessee, UNITED STATES

## Fungal diseases: A real threat to public health

Human fungal diseases differ fundamentally from other infections in diverse ways. As eukaryotic pathogens, fungi share many similarities with their host cells, which impairs the development of antifungal compounds. Fungal tropism is highly variable, as pathogens infect a wide range of cell types. A single fungal pathogen can infect multiple tissues in the same patient (depending on the host’s immunological status) and can undergo morphogenic shifts during infection. Fungi are still underappreciated as major pathogens by both the public and public health officials. Diseases caused by protozoa, bacteria, and viruses have been recognized as important public health issues for centuries. For instance, syphilis, influenza, and Chagas disease have been documented for over 100 years [1], while invasive mycoses were only widely acknowledged as medically important pathogens in the 1980s [2].

Viral diseases of major population impact (such as smallpox, influenza and—more recently—dengue, Zika, Chikungunya, and coronavirus) have affected millions of people with significant effects on human health in developed, developing, and less developed nations [3,4]. These conditions have boosted the generation of knowledge, which has led to the eradication of smallpox [5], the wide availability of effective vaccines [6], and the development of diagnostic and preventive tools against influenza [7] and, more recently, Zika [8]. Bacterial diseases have profoundly impacted human health at different times in history, and although the phenomenon of antimicrobial resistance is a matter of extreme concern, there are several effective tools for the prevention, treatment, and diagnosis of bacterial infections [9]. Human parasitosis have been recognized to negatively impact public health in different parts of the globe for decades, which has stimulated the ongoing development of vaccines, new drugs, and diagnostic tests for malaria, sleeping sickness, leishmaniasis, filariasis, and Chagas disease [10]. Fungal infections, however, are part of a different scenario. These diseases, for most of recorded history as well as the majority of the last century, have been rare or had a low impact on human health.

The increase in the number of immunocompromised patients, some of whom are highly susceptible to fungal infections, has totally changed this picture. The invasive diseases caused by fungi, the so-called systemic mycoses, profoundly impact human health. Moreover, the Global Action Fund for Fungal Infections (GAFFI) also highlights the devastating impact of focal fungal diseases in individuals who often have intact immune systems. GAFFI estimates that more than 1 million eyes go blind each year due to fungal keratitis [11]. Nearly one billion people have skin mycoses, which makes this disease only slightly less common on the planet than headaches and dental caries. Fungal spores contribute to significant reactive airway diseases in over 10 million individuals. In total, the GAFFI estimates that over 300 million people of all ages suffer from a serious fungal infection each year globally [11]. Notably, over 1.5 million of these individuals are estimated to die from their fungal disease [12].

Individual fungal diseases have profound impacts on human health. Around 220,000 new cases of cryptococcal meningitis occur worldwide each year, resulting in 181,000 deaths concentrated in sub-Saharan Africa [13]. More than 400,000 people develop *Pneumocystis* pneumonia annually and die without access to therapy [11]. In Latin America, histoplasmosis is one of the most common opportunistic infections among people living with HIV/AIDS, and approximately 30% of patients diagnosed with histoplasmosis in that region die from this disease [12]. Morbidity rates linked to fungal infections also represent an important health issue. For example, diseases such as chromoblastomycosis and eumycetoma lead to destructive deformations and debilitating conditions of the subcutaneous tissues, skin, and underlying bones, which result in social exclusion [14].

## AIDS and opportunistic fungal diseases: Problem solved or current threat?

Along with patients on anticancer therapies and other immunosuppressive medications, individuals with advanced HIV have dramatically contributed to the excess numbers of deaths due to fungal diseases. The implementation of new therapeutic strategies has had an unquestionably positive impact on the health of individuals with HIV and, as a result, AIDS-related deaths have fallen by more than 50% since their peak in 2004. The global number of people living with HIV ranged from 32.7 million to 44 million in 2018. In this group, up to 23.3 million people had access to antiretroviral therapy. In 2017, about 1.7 million new HIV infections were reported, and about 770,000 people died from this condition. It is noteworthy that up to 75 million people have been infected with HIV since the start of the pandemic, resulting in approximately 32 million AIDS-related deaths [15]. Hence, there remain large numbers of individuals who are not in care or whose immune systems are compromised by HIV. These compromised HIV-infected individuals, particularly those with CD4+ cell counts less than 200/mm^3^, are at high risk for invasive fungal diseases. Thus, the spread or control of AIDS is directly linked to the impact of invasive mycoses on public health.

Tuberculosis remains the leading cause of death among people living with HIV, accounting for about 1 in 3 AIDS-related deaths [15]. By the end of 2016, 1.2 million people living with HIV developed tuberculosis. However, it is important to reinforce that invasive mycoses have a similarly close relationship to AIDS. According to the Centers for Disease Control and Prevention (CDC), fungi are among the leading causes of opportunistic infections affecting patients with HIV/AIDS [16]. Even with the increasing availability of anti-HIV treatment in less developed countries, fungal infections, particularly cryptococcosis and histoplasmosis [12,13], are still a major problem for people living with HIV/AIDS. For example, meningitis caused by the genus *Cryptococcus* is (after tuberculosis) the second leading cause of death in people living with HIV [13]. Importantly, cryptococcal meningitis is a brain infection that, if left untreated, results in an agonizing death for people living with HIV [17].

## Systemic mycoses are neglected diseases

Despite their alarming impact on human health, fungal diseases have been continually neglected over the years. According to Molyneux [18], neglected tropical diseases have particular characteristics. Firstly, they afflict the poorest people without access to safe drinking water, sanitation, and basic health services. Secondly, they are usually chronic and slowly developing, becoming progressively worse if left undiagnosed and untreated. The damage these diseases cause can be irreversible. Finally, neglected tropical diseases can cause severe pain and disability throughout life, with long-term consequences for patients and families of the affected person. People with neglected tropical diseases are often stigmatized and socially excluded, which can affect their mental health. High-income groups are rarely affected.

The number of diseases that meet the above criteria is regrettably higher than would be expected for the second decade of the current millennium. This number, however, is underestimated, as several important syndromes fit these criteria but are not formally recognized as such, including systemic mycoses. Indeed, most high-mortality mycoses remain ignored by public health authorities and decision-makers. The financial support for fungal disease research is incredibly lower than the funding available for other infectious diseases that cause similar mortality [19,20]. For instance, for each human individual dying from malaria, US$1,315 are invested in research and development. Investment per death corresponds to US$334 for tuberculosis, US$276 for diarrheal diseases, and only US$31 for cryptococcal meningitis [19]. Still, there is no clear recognition of the importance of fungal diseases by international health agencies. For example, the World Health Organization (WHO) has recently included mycetoma, chromoblastomycosis, and “other deep mycoses” in the list of neglected tropical diseases [21], but specific information on WHO plans to combat fungal diseases is not yet available. Research on histoplasmosis, paracoccidioidomycosis, and sporotrichosis receives negligible funding [19]. Although these diseases are associated with high rates of mortality or the generation of conditions that hinder the performance of professional functions and social integration [14], none of them has been formally recognized as neglected diseases by WHO.

According to Morel [22], neglected diseases persist due to failures in science, market, and public health. Science failures occur when there is insufficient knowledge on the pathophysiology of infectious agents and the host response. Market failures are usually observed in diseases against which medicines or vaccines exist but at a prohibitive cost. Finally, public health failures occur in syndromes against which low cost or even free prophylactic tools and medicines are available but their use is limited by poor logistics and lack of governmental support.

Fungal diseases are clearly affected by the 3 types of failures described above. In this field, there has been a significant failure in science compared to diseases of medical importance recognized for decades or centuries, as previously mentioned. Of course, significant gaps in knowledge generation rates exist. Fungal infections consist of pathogenic processes triggered by eukaryotic microorganisms, which hinders the development of drugs that are toxic to the pathogen without affecting host tissues.

The fact that there are no licensed antifungal vaccines underscores another clear failure in science. Similarly, reliable diagnostic methods are available for a very limited number of mycoses [23], and therapeutic options are restricted to a few classes of drugs that too frequently are associated to both intrinsic and acquired resistance [24], toxic, and expensive [25]. In fact, innovative tools to combat invasive mycoses are rare and of slow development. For illustration, the most recently developed antifungals (echinocandins) were approved for clinical use in 2002 [26], reinforcing a major science failure in the area. It is noteworthy that this class of drugs is ineffective against various high-mortality mycoses [25].

Market failures have a profound impact on the control of fungal diseases. The deadliest fungal infections affect neglected populations, which results in a reduced market for drug commercialization and lack of interest from the pharmaceutical sector in the development of medicines, vaccines, and diagnostic tests for human mycoses. The main drug historically used for the treatment of severe disseminated mycoses is amphotericin B (AmB), whose discovery dates to 1955 [27], and it remains the standard first-line medication for certain fungal infections, such as cryptococcal meningitis. AmB formulations used for invasive fungal infections vary greatly in efficacy, safety, and cost. Conventional formulations are usually affordable but include significant side effects. The most effective and least toxic formulation is liposomal AmB, which can generate costs of up to US$100,000 per patient in different parts of the globe, including developing countries [28].

Liposomal AmB is highly effective when used in combination with other drugs. This pharmaceutical preparation was recommended by WHO as the preferred treatment for cryptococcal meningitis [29]. However, the high prices and unavailability of liposomal AmB in several countries have created major barriers to access to the most recommended treatment—as recognized by WHO itself—in developing countries. Liposomal AmB is registered and available for use (at high cost) in only 6 of 116 developing countries where fungal meningitis is a public health problem [11]. Prices are impeditive in many countries, revealing an unquestionable market failure.

Public health failures also impact fungal diseases negatively. According to GAFFI [11], several major antifungals are not available or registered in various regions where fungal diseases are most lethal. 5-Fluorocytosine, a low-cost antimetabolite that is beneficial to a number of patients with systemic mycoses when used in combination with other antifungal drugs, is not available and/or registered in many countries, including those highly affected by systemic mycoses [11]. Given the intrinsic difficulties and high costs of drug development and the evident market and public health failures in this field, it is more realistic and impactful to make rational use of the diagnostic and antifungal tests already available to minimize the number of deaths caused by fungal diseases. In a recent study, Denning [30] proposed actions to reduce deaths from fungal diseases on the basis of currently available diagnostic tests and generic antifungals. Assuming that diagnostic tests would be properly applied and that antifungal therapy would be administered promptly and following current international guidelines, it was estimated that by 2020 annual deaths from cryptococcal meningitis could fall from 180,000 to 70,000. Deaths due to *Pneumocystis* pneumonia would fall from 400,000 annually to 162,500. The 80,000 annual deaths attributable to disseminated histoplasmosis could be reduced by 60%. Annual deaths due to chronic pulmonary aspergillosis (56,288) could fall by 33,500. These actions would thus result in a total of 1 million lives saved over 5 years. Of course, the effective implementation of AIDS control and prevention campaigns in areas lacking such programs would also positively impact the reduction in deaths caused by fungal infections. Such actions have the potential to minimize a clear public health failure on the basis of the use of existing tools for diagnosing and treating invasive mycoses.

## Present and future problems: The unknown

The epidemiology of fungal diseases is dynamic, and changes are difficult to predict. In 2012, the CDC reported an outbreak of fungal infections of the central nervous system that occurred among patients who received epidural or paraspinal injections of methylprednisolone. The majority of affected patients had meningitis caused by an extremely rare cause of fungal disease, namely *Exserohilum rostratum* [31]. This fungus is an example of an unexpected, emergent fungal disease, and it reinforced the perception of the pathogenic potential inherent in the fungal kingdom. The *E*. *rostratum* outbreak killed over 60 people out of 750 infected patients [32].

There is also a growing perception that climate change directly impacts the ability of fungi to cause damage to the human host. Recently, the multiresistant pathogen *Candida auris* has emerged as a serious global threat to human health, causing infections resistant to all major classes of antifungal drugs in immunocompromised patients [33]. *C*. *auris* differs from most other *Candida* species in several aspects. As recently reviewed by Lockhart, *C*. *auris* colonizes the skin rather than the gastrointestinal tract and is extremely resilient in the environment [34]. This resiliency has led to the fungus being associated with healthcare outbreaks, which have been exceedingly difficult to control due to the remarkable difficulty in eradicating the fungus from both patients and the environment [33]. Also of great concern, antimicrobial resistance in *C*. *auris* is more common than susceptibility to antifungals [34]. The spread of *C*. *auris* disease is linked to clonal isolates recovered from India, Venezuela, and South Africa between 2012 and 2015. Widespread use of antifungal drugs has been suggested as a determining factor for the emergence of *C*. *auris* [35]. Another hypothesis for the emergence of *C*. *auris* suggests that the fungus has recently acquired the virulence characteristics required to cause damage to human hosts. Although these explanations cannot be ruled out, it is unlikely that these changes occurred simultaneously on 3 continents. In this sense, it has recently been proposed that isolates of *C*. *auris* adapted to the human body temperature through selection from high-temperature regions [35]. Thus, this would be the first example of a novel human fungal pathogen that emerged as a result of global warming, which would explain several of its pathogenic characteristics. This observation demonstrates an important link between climate change and infectious diseases. Importantly, new threats to human health might still occur through climate adaptation mechanisms of zoonotic fungi, as proposed for *C*. *auris*.

Diseases that are known for decades still raise concerns. The city of Rio de Janeiro, Brazil, currently faces the largest sporotrichosis epidemic in history from a species, *Sporothrix brasiliensis*, that emerged locally [36]. Paracoccidioidomycosis is still one of the most important systemic mycoses in Latin America and the leading cause of mycosis mortality in immunocompetent individuals in Brazil [37]. Globally, the latest estimates suggest an annual occurrence of approximately 3 million cases of chronic pulmonary aspergillosis, over 200,000 cases of cryptococcal meningitis, 700,000 cases of invasive candidiasis, 500,000 cases of *Pneumocystis jirovecii* pneumonia, 250,000 cases of invasive aspergillosis, 100,000 cases of histoplasmosis, over 10 million cases of fungal asthma, and 1 million cases of fungal keratitis [12].

## The need for improved diagnosis of fungal infections

Early diagnosis of mycoses is decisive to efficient therapy. Common methods for the laboratory diagnosis of fungal infections include direct microscopic examination of human or animal samples, histopathology, microbial culture, antigen detection, serology, and, in a few cases, molecular tests [38]. These tests are relatively efficient at identifying well-known pathogens as causative agents of human and animal syndromes. Difficulties are nevertheless present as fungi typically reproduce slowly, and culture methods may take as long as a month to identify common species such as *Histoplasma* sp. Moreover, susceptibility testing to guide clinicians is also problematic, and breakpoints are not available for several important human pathogenic fungi, including *C*. *auris* [39]. Moreover, as reviewed by Wickes and Wiederhold [23], detection of less frequently encountered fungi is considerably more complex because routine clinical laboratories may lack the expertise and appropriate equipment to identify pathogenic agents. In fact, a recent survey involving 129 major laboratory centers in 24 countries of Latin America and the Caribbean revealed that only 9% of these centers appear to potentially meet the minimum European Confederation of Medical Mycology standards for fungal laboratory diagnostics [40]. Furthermore, in the national laboratories of developing countries, there is an enormous demand for the diagnostics of other infectious (quite often epidemic) diseases, and the lack of trained personnel is an additional limitation.

The impact of this condition on human health is clear. For example, *Exserohilum* diseases before the 2012 United States outbreak manifested as rare systemic, cutaneous, corneal, and subcutaneous infections [41]. As the causative agent of the meningitis outbreak, *E*. *rostratum* was identified 1 month after the first meningitis case was reported, when the CDC announced that *E*. *rostratum* was recovered from unopened vials of steroid injections [42]. Similar problems occurred with necrotizing mucormycosis, a devastating complication of wounds caused by *Apophysomyces* sp., *Saksenaea* sp., and *Lichtheimia* [43]. Late laboratory diagnosis of cases of necrotizing mucormycosis caused by *Apophysomyces trapeziformis* resulted in 5 deaths in Missouri in 2011 [44]. Addressing the epidemic of *C*. *auris* has also been impeded by inherent deficiencies with classical laboratory methods utilized by many clinical laboratories as well as public health institutions [39]. These cases reinforce the notion that tests rapidly identifying infecting fungi have the potential to impact the course of fungal diseases beneficially.

## Funding for research and innovation in fungal diseases

Funding for research on fungal diseases is unquestionably small compared to funding available for other infectious diseases that cause similar mortality [19,20]. As an illustration, funding for research on cryptococcal meningitis, the fifth deadliest infectious disease, receives 4.3-fold less funding than the disease caused by the bacterial pathogen *Neisseria meningititis* [19]. Of concern, reports submitted between 2008 and 2017 on funding for neglected diseases show that cryptococcal meningitis was the only measurably funded fungal disease, accounting for 0.5% of the total invested [45]. Tuberculosis, for comparison, had a 34-fold higher investment. Other fungal diseases were not even included in these reports. Specifically, neglected mycoses of unquestionable clinical importance—such as paracoccidioidomycosis, mycetoma, sporotrichosis, and chromoblastomycosis—have not even been mentioned in the report, suggesting that these research areas have received negligible funding. These observations were fully confirmed by direct analysis of scientific articles declaring financial support from major international agencies with a history of supporting neglected disease research [45].

Reduced support for research and innovation in fungal diseases impacts knowledge generation directly. For example, tuberculosis and malaria were the focus of 8,827 and 5,687 scientific articles published in 2017, respectively. Fungal diseases, on the other hand, were much less investigated, with 213 articles on cryptococcosis, 80 on paracoccidioidomycosis, 51 on chromoblastomycosis, 53 on mycetoma, and 56 on sporotrichosis produced in the same period [45]. These numbers are probably linked to alarming facts, such as the aforementioned lack of vaccines capable of preventing fungal disease, less effective diagnostics, and a dearth of antifungal drugs in development.

## Perspectives

There are ongoing initiatives to develop antifungal vaccines and drugs with the potential to control invasive mycoses [46]. However, the distance between promising laboratory results and the translation of knowledge into benefits to the general population is unquestionably long. In fungal diseases, this distance is apparently longer, considering the lack of investment in science and technology in association with the science, market, and public health failures discussed here. The situation is even more complex if one considers the emergence of multiresistant and still largely unknown pathogens such as *C*. *auris*. The impact of emerging infections of this nature on human health is still hard to predict, but, as *C*. *auris* is now spread across the globe, the reality is that such infections can lead to significant morbidity and mortality as well as have vast economic consequences. Thus, it seems clear that public health authorities and decision-makers need to more thoughtfully and closely consider invasive fungal diseases as a real and contemporary problem to avoid disasters historically observed in other models of infectious diseases. The fact that fungal diseases are not spread at the same rate as other microbial transmissible diseases causing epidemics does not mean they are less relevant in terms of the number of attained individuals. Furthermore, the fact that they are less studied represents an enormous risk given the new potential threats as a consequence of environmental deterioration and global warming.

Realistic discussions about how prevention, diagnosis, and control of fungal diseases will improve outcomes demand a separation between concrete actions using currently available tools and future preventive actions. Of course, prophylactic actions against as yet unknown conditions are complex and difficult to develop, but the recent history of emerging fungal diseases reveals a clear need for knowledge generation on fungal pathogens. The attention to emerging fungal pathogens is important because even in the case they do not cause disease to humans due to new and as yet unknown zoonosis, they can affect animal health with an impact in the economy. Also, they can affect wild animals with an unpredictable ecological impact on biodiversity. Stimulating basic science and innovative activities in the area is therefore essential to reduce the impact of poorly known or yet unknown fungal diseases on human health.

On the basis of currently available therapeutic and diagnostic tools, short- and medium-term impact actions also need to be implemented. For example, in 2019, WHO reinforced the need to use the *Histoplasma capsulatum* antigen detection test to diagnose histoplasmosis [47]. This test allows the diagnosis of the disease in more than 85% of patients within 48 hours, which would hasten the implementation of lifesaving antifungal therapy. Without proper diagnosis, patients are usually treated for tuberculosis, which has similar clinical symptoms. Under these conditions, patients usually die within 1 to 3 weeks. It is estimated that 48,000 lives could be saved over 5 years if appropriate diagnosis and treatment approaches are implemented for histoplasmosis.

The above examples illustrate the complexity behind well-known and still poorly known fungal diseases. In both scenarios, concrete actions can be implemented, Support for basic research and technological development is obviously important, but making health professionals and decision-makers aware of the profound and ongoing impact of fungal diseases on human health is essential. The current situation, however, raises serious concerns, considering the funding limitations in the area and lack of public programs for prevention and control of fungal diseases. The high incidence of invasive mycoses in AIDS patients and the recent examples of *C*. *auris* and *E*. *rostratum* demonstrate that, without game-changing actions, the perspective on how fungal diseases will impact human health in the coming decades is extremely negative.

## References

[1] Brachman PS. Infectious diseases—Past, present, and future. International Journal of Epidemiology. 2003 10.1093/ije/dyg282 14559728

[2] Nucci M, Marr KA. Emerging Fungal Diseases. Clin Infect Dis. 2005; 10.1086/432060 16028162

[3] Woolhouse MEJ, Howey R, Gaunt E, Reilly L, Chase-Topping M, Savill N. Temporal trends in the discovery of human viruses. Proc R Soc B Biol Sci. 2008; 10.1098/rspb.2008.0294 18505720 PMC2475551

[4] Woolhouse M, Scott F, Hudson Z, Howey R, Chase-Topping M. Human viruses: Discovery and emeraence. Philos Trans R Soc B Biol Sci. 2012; 10.1098/rstb.2011.0354 22966141 PMC3427559

[5] Strassburg MA. The global eradication of smallpox. AJIC Am J Infect Control. 1982; 10.1016/0196-6553(82)90003-7 7044193

[6] CDC. Recommended Vaccines by Age [Internet]. 2016 Available from: https://www.cdc.gov/vaccines/vpd/vaccines-age.html. [cited 2019 Sep 4].

[7] Marzoratti L, Iannella HA, Gómez VF, Figueroa SB. Recent advances in the diagnosis and treatment of influenza pneumonia. Curr Infect Dis Rep. 2012; 10.1007/s11908-012-0257-5 22477036 PMC3342505

[8] Barrett ADT. Current status of Zika vaccine development: Zika vaccines advance into clinical evaluation /692/308/153 /631/326/590/1883 perspective. npj Vaccines. 2018; 10.1038/s41541-018-0061-9 29900012 PMC5995964

[9] Aminov RI. A brief history of the antibiotic era: Lessons learned and challenges for the future. Front Microbiol. 2010; 10.3389/fmicb.2010.00134 21687759 PMC3109405

[10] Sacks DL. Vaccines against tropical parasitic diseases: A persisting answer to a persisting problem. Nature Immunology. 2014 10.1038/ni.2853 24747701 PMC4814932

[11] GAFFI. Global Fund for Fungal Infections [Internet]. 2018. Available from: https://www.gaffi.org. [cited 2018 Aug 8]

[12] Bongomin F, Gago S, Oladele RO, Denning DW. Global and multi-national prevalence of fungal diseases—estimate precision. Journal of Fungi. 2017 10.3390/jof3040057 29371573 PMC5753159

[13] Rajasingham R, Smith RM, Park BJ, Jarvis JN, Govender NP, Chiller TM, et al Global burden of disease of HIV-associated cryptococcal meningitis: an updated analysis. Lancet Infect Dis. 2017;17: 873–881. 10.1016/S1473-3099(17)30243-8 28483415 PMC5818156

[14] Queiroz-Telles F, Fahal AH, Falci DR, Caceres DH, Chiller T, Pasqualotto AC. Neglected endemic mycoses. The Lancet Infectious Diseases. 2017 10.1016/S1473-3099(17)30306-7 28774696

[15] UNAIDS. Global HIV & AIDS statistics—2019 fact sheet [Internet]. 2019.

[16] CDC. Fungal Diseases [Internet]. 2017 Available from: https://www.cdc.gov/fungal/infections/hiv-aids.html. [cited 2019 Sep 3].

[17] Colombo AC, Rodrigues ML. Fungal colonization of the brain: Anatomopathological aspects of neurological cryptococcosis. An Acad Bras Cienc. 2015;87: 1293–1309. 10.1590/0001-3765201520140704 26247147

[18] Molyneux David. Neglected tropical diseases. Community Eye Heal. 2013;26: 21–24. PMC3756642 24023397

[19] Rodrigues ML, Albuquerque PC. Searching for a change: The need for increased support for public health and research on fungal diseases. PLoS Negl Trop Dis. 2018;12: 1–5. 10.1371/journal.pntd.0006479 29902170 PMC6001980

[20] Rodrigues ML. Funding and Innovation in Diseases of Neglected Populations: The Paradox of Cryptococcal Meningitis. PLoS Negl Trop Dis. 2016;10 10.1371/journal.pntd.0004429 26964103 PMC4786104

[21] WHO. Neglected tropical diseases [Internet]. 2017. Available from: https://www.who.int/neglected_diseases/diseases/en/. [cited 2019 Sep 3].

[22] Morel CM. [Innovation in health and neglected diseases]. Cad Saude Publica. 2006; doi: /S0102-311X2006000800001 16832524 10.1590/s0102-311x2006000800001

[23] Wickes BL, Wiederhold NP. Molecular diagnostics in medical mycology. Nature Communications. 2018 10.1038/s41467-018-07556-5 30510235 PMC6277409

[24] Robbins N, Caplan T, Cowen LE. Molecular Evolution of Antifungal Drug Resistance. Annu Rev Microbiol. 2017;71: 753–775. 10.1146/annurev-micro-030117-020345 28886681

[25] Mourad A, Perfect JR. The war on cryptococcosis: A review of the antifungal arsenal. Memorias do Instituto Oswaldo Cruz. 2018 10.1590/0074-02760170391 29513785 PMC5851043

[26] Denning DW. Echinocandins: a new class of antifungal. J Antimicrob Chemother. 2002; 10.1093/jac/dkf045 12039879

[27] Oura M, Sternberg TH, Wright ET. A new antifungal antibiotic, amphotericin B. Antibiot Annu. 1955;3:566–573. 13355328

[28] Borba HHL, Steimbach LM, Riveros BS, Tonin FS, Ferreira VL, Bagatim BA de Q, et al Cost‐effectiveness of amphotericin B formulations in the treatment of systemic fungal infections. Mycoses. 2018;12 June 20. 10.1111/myc.12801 29893450

[29] WHO. Guidelines for the diagnosis, prevention and management of cryptococcal disease in HIV-infected adults, adolescents and children. Supplement to the 2016 consolidated guidelines on the use of antiretroviral drugs for treating and preventing HIV infection. 2018 pp. 1–45. Available from: https://www.who.int/hiv/pub/guidelines/cryptococcal-disease/en/. [cited 2019 Sep 3]. 30285342

[30] Denning DW. Minimizing fungal disease deaths will allow the UNAIDS target of reducing annual AIDS deaths below 500 000 by 2020 to be realized. Philos Trans R Soc B Biol Sci. 2016; 10.1098/rstb.2015.0468 28080991 PMC5095544

[31] Smith RM, Schaefer MK, Kainer MA, Wise M, Finks J, Duwve J, et al Fungal infections associated with contaminated methylprednisolone injections. N Engl J Med. 2013; 10.1056/NEJMoa1213978 23252499

[32] CDC. Multistate Outbreak of Fungal Meningitis and Other Infections–Case Count [Internet]. 2015 Available from: https://www.cdc.gov/hai/outbreaks/meningitis-map-large.html. [cited 2019 Sep 4].

[33] Clancy CJ, Nguyen MH. Emergence of candida auris: An international call to arms. Clinical Infectious Diseases. 2017 10.1093/cid/ciw696 27989986

[34] Lockhart SR. Candida auris and multidrug resistance: Defining the new normal. Fungal Genetics and Biology. 2019 10.1016/j.fgb.2019.103243 31228646 PMC12012538

[35] Casadevall A, Kontoyiannis DP, Robert V. On the Emergence of Candida auris : Climate Change, Azoles, Swamps, and Birds. MBio. 2019; 10.1128/mbio.01397-19 31337723 PMC6650554

[36] Gremião IDF, Miranda LHM, Reis EG, Rodrigues AM, Pereira SA. Zoonotic Epidemic of Sporotrichosis: Cat to Human Transmission. PLoS Pathog. 2017 10.1371/journal.ppat.1006077 28103311 PMC5245785

[37] Giacomazzi J, Baethgen L, Carneiro LC, Millington MA, Denning DW, Colombo AL, et al The burden of serious human fungal infections in Brazil. Mycoses. 2016;59: 145–150. 10.1111/myc.12427 26691607

[38] Backx M, White PL, Barnes RA. New fungal diagnostics. British Journal of Hospital Medicine. 2014 10.12968/hmed.2014.75.5.271 25040272

[39] Kordalewska M, Perlin DS. Identification of Drug Resistant Candida auris. Front Microbiol. 2019;10:1918 10.3389/fmicb.2019.01918 31481947 PMC6710336

[40] Falci DR, Pasqualotto AC. Clinical mycology in Latin America and the Caribbean: A snapshot of diagnostic and therapeutic capabilities. Mycoses. 2019;62: 368–373. 10.1111/myc.12890 30614600

[41] Katragkou A, Pana ZD, Perlin DS, Kontoyiannis DP, Walsh TJ, Roilides E. Exserohilum infections: Review of 48 cases before the 2012 United States outbreak. Med Mycol. 2014; 10.1093/mmy/myt030 24682112

[42] Larone DH, Walsh TJ. Exserohilum rostratum: Anatomy of a national outbreak of fungal meningitis. Clin Microbiol Newsl. 2013; 10.1016/j.clinmicnews.2013.08.007

[43] Walsh TJ, Hospenthal DR, Petraitis V, Kontoyiannis DP. Necrotizing Mucormycosis of Wounds Following Combat Injuries, Natural Disasters, Burns, and Other Trauma. J Fungi. 2019; 10.3390/jof5030057 31277364 PMC6787580

[44] Fanfair RN, Benedict K, Bos J, Bennett SD, Lo YC, Adebanjo T, et al Necrotizing cutaneous mucormycosis after a Tornado in Joplin, Missouri, in 2011. N Engl J Med. 2012; 10.1056/NEJMoa1204781 23215557

[45] Rodrigues ML, Albuquerque PC. Searching for a change: The need for increased support for public health and research on fungal diseases. PLoS Negl Trop Dis. 2018;12 10.1371/journal.pntd.0006479 29902170 PMC6001980

[46] McCarthy MW, Walsh TJ. Drug development challenges and strategies to address emerging and resistant fungal pathogens. Expert Rev Anti Infect Ther. 2017;15: 577–584. 10.1080/14787210.2017.1328279 28480775

[47] WHO. Second WHO Model List of Essential In Vitro Diagnostics. 2019.

